# High-*T_g_* Vat Photopolymerization Materials Based on In Situ Sequential Interpenetrating Polymer Networks of Maleimide and Cyanate Ester Monomers

**DOI:** 10.3390/polym17233179

**Published:** 2025-11-29

**Authors:** Anh Fridman, Nicolas J. Alvarez, Giuseppe R. Palmese

**Affiliations:** 1 Department of Chemical and Biological Engineering, Drexel University, Philadelphia, PA 19146, USA; 2 Department of Chemical Engineering, Rowan University, Glassboro, NJ 08028, USA

**Keywords:** vat photopolymerization, interpenetrating network, high-performance, shrinkage

## Abstract

There are limited material choices for vat photopolymerization additive manufacturing processes that offer high dimensional accuracy. Acrylates and epoxies are commonly used, but their thermal properties are not suitable for applications requiring high-temperature performance. A possible solution is the use of high-performance thermosets, such as maleimide and cyanate ester, which are cured at high temperatures. Still, their use in vat photopolymerization methods has been limited due to the high temperature required. In this work, a photocurable formulation composed of multimaleimide monomers, a reactive diluent, and a cyanate ester was developed to improve thermal and mechanical properties and reduce cure shrinkage due to density changes during processing. In situ sequential interpenetrating polymer networks (IPNs) were investigated, in which the copolymerization of multimaleimide and a diluent occurs during printing, yielding a cyanate ester-swollen network with a sub-room-temperature glass transition temperature (*T_g_*). The polymerization of the cyanate ester occurs during a high-temperature post-printing step. The resulting materials have a *T_g_* above 250 °C (peak in the loss modulus), good fracture toughness (GIc of 100 J/m^2^), and overall cure shrinkage of less than 6%, with 1–2% occurring during the high-temperature post-curing step.

## 1. Introduction

Additive manufacturing has expanded rapidly in recent years due to advantages such as design freedom, product customization, reduced waste, and cost efficiency [1]. Among the available approaches, vat photopolymerization is widely used because it can generate parts with high spatial resolution. However, the materials compatible with this technique are restricted by photochemical requirements and are typically limited to acrylates, methacrylates, and epoxies [2]. Although these systems cure efficiently, their thermal performance is inadequate for applications that demand high-temperature stability. As a result, high-performance thermosets that require elevated curing temperatures are rarely incorporated into SLA or DLP processes. Recent work has demonstrated alternative strategies, including direct ink writing of cyanate ester composites [3] and UV-assisted printing of bismaleimides [4], but these methods fall outside standard vat photopolymerization workflows. Efforts to print aromatic polyimides show that materials with high thermal resistance can be processed, but they often suffer from substantial shrinkage and diminished mechanical properties [5]. Reports on the *T_g_* of SLA/DLP materials remain limited, with the *T_g_* typically falling between 80 and 200 °C (tan δ) [4,6,7].

Parts produced by SLA or DLP can also accumulate significant residual stresses because thermoset polymerization involves volumetric shrinkage as crosslinks form [8,9]. During layer-by-layer printing, this shrinkage occurs while each layer is constrained by the previously cured material and the build platform, which can promote warpage, delamination, or local damage, ultimately affecting mechanical performance [10]. The magnitude of cure shrinkage is strongly dependent on molecular structure, and common acrylate and methacrylate systems typically exhibit 5–10% density change during polymerization [2]. Another challenge in vat photopolymerization of high-*T_g_* systems is that cure reactions at room temperature are restricted by vitrification: once the *T_g_* approaches the reaction temperature, diffusion slows and conversion levels off [11]. Lange et al. further showed that substantial shrinkage occurring near vitrification can produce elevated residual stresses due to the simultaneous rise in the modulus [12]. These factors motivate approaches that can increase conversion, suppress vitrification during printing, and limit shrinkage-induced stress.

**Figure 1:**
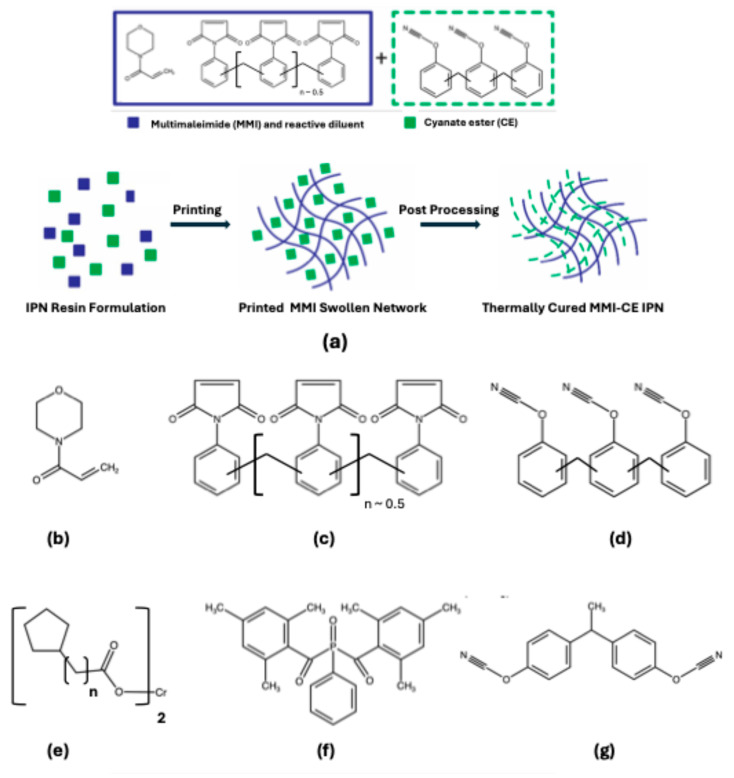
(**a**) Schematic of in situ sequential IPN using free-radical cure during printing, followed by high-temperature cyanate ester cure post-processing. Chemical structures of resin components used in this work: (**b**) acryloylmorpholine, (**c**) poly(phenylmethane) maleimide (n = 0.5), (**d**) novolac-based cyanate ester, (**e**) copper (II) napthenate, (**f**) phenyl bis(2,4,6 trimethylbenzoyl) phosphine oxide, (**g**) bisphenol E cyanate ester.

This work focuses on in situ sequential interpenetrating polymer networks (IPNs) as a strategy to address these limitations while enabling the use of high-temperature materials. IPNs have been investigated for vat photopolymerization, most commonly through combinations of free-radical acrylate polymerization and cationic epoxy polymerization [13,14]. Two-stage acrylate–epoxy systems—where the acrylate network is formed photochemically and the epoxy network forms thermally—have also been explored in SLA [7]. Our study retains the advantages of two-stage processing but applies it to monomers traditionally cured at high temperature. Multimaleimide (MMI) and cyanate ester (CE) resins have desirable thermal resistance, good dielectric properties, and low moisture uptake [15,16,17], but these attributes come with cure temperatures that are incompatible with standard vat photopolymerization workflows. Neat MMI networks are also characteristically brittle, although previous studies have shown that forming IPNs with cyanate esters can substantially increase toughness [18,19,20]. To formulate an MMI–CE system compatible with SLA or DLP, a reactive diluent capable of copolymerizing with the maleimide groups is required. Prior work has shown that bismaleimide can copolymerize with acryloylmorpholine through an electron-donor/acceptor mechanism under UV or electron-beam irradiation when appropriate photoinitiators are present [19,21]. In our approach, a sequential IPN is created (Figure 1): MMI and acryloylmorpholine first copolymerize during printing to form a CE-swollen network with a *T_g_* below room temperature, and the cyanate ester polymerizes subsequently during a high-temperature post-cure. This design delays vitrification, allowing higher double-bond conversion in the rubbery state and lowering overall cure shrinkage owing to the low-shrinkage nature of CE resins [17].

## 2. Experimental Section

### 2.1. Materials

Poly(phenylmethane) maleimide was supplied by Miki Sangyo (Parsippany, NJ, USA). Acryloylmorpholine (*AMP*) was obtained from TCI Chemicals. Two cyanate ester monomers were used in this study: a novolac-based cyanate ester (AroCy XU371) and a bisphenol E cyanate ester (AroCy L-10), both provided by Huntsman (Houston, TX, USA). Copper(II) naphthenate was purchased from Strem Chemicals (Newburyport, MA, USA), and phenyl bis(2,4,6-trimethylbenzoyl) phosphine oxide (PPO) was obtained from Sigma Aldrich (St. Louis, MO, USA). All materials were used as received. The chemical structures of the resin components are shown in Figure 1.

### 2.2. Resin Preparation

*PMMI*-*AMP* resins: Poly(phenylmethane) maleimide (*PMMI*) was dissolved in 4-acryloylmorpholine (*AMP*) at 70 °C to prepare mixtures containing various *PMMI* weight fractions. After complete dissolution, phenyl bis(2,4,6-trimethylbenzoyl) phosphine oxide (PPO) was added at 0.5 wt% relative to the total resin mass and mixed until fully incorporated.

IPN resins: For the IPN formulations, copper(II) naphthenate was first blended into novolac-based cyanate ester (n-CE) at 70 °C to achieve a catalyst concentration of 600 ppm Cu^2+^. This catalyzed CE resin was then combined with the 30 wt% *PMMI*-in-*AMP* mixture at varying mass ratios. PPO was again incorporated at 0.5 wt% relative to the total resin mass. Preparation of IPN resins using bisphenol E cyanate ester (bisE-CE) followed the same procedure, substituting bisE-CE for n-CE.

### 2.3. Experimental Procedures and Characterizations

Resin viscosity and density: Viscosity measurements were conducted on an AR2000EX rheometer (TA Instruments, New Castle, DE, USA) using a 40 mm, 1° cone-and-plate geometry. All tests were performed at 25 °C over shear rates ranging from 0.01 to 100 s^−1^. Resin densities were determined at 25 °C using an Anton Paar DMA800 density meter (Ashland, Wilmington, DE, USA).

Printing and post-curing procedures: All formulations were printed on an Anycubic Photon masked-projection DLP printer (Shenzhen, China) equipped with a 405 nm LED light source. The light intensity at the LCD surface was 0.51 mW/cm^2^ (measured using an ILT 2400 sensor (International Light Technologies (ILT), Peabody, MA, USA) operating from 275 to 450 nm). A layer thickness of 100 μm was used for all prints, with exposure times selected based on the working curves discussed in Section 3.1. After printing, samples were UV-post-cured in a Formlabs UV oven (Formlabs, Somerville, MA, USA) at 80 °C for 1 h. *PMMI*-*AMP* systems were then thermally post-cured at 120 °C for 2 h, 180 °C for 3 h, 220 °C for 4 h, and 280 °C for 1 h. IPN systems were thermally post-cured at 180 °C for 3 h, 220 °C for 4 h, and 280 °C for 1 h.

Dynamic Mechanical Analysis: Glass transition temperatures (*T_g_*) were measured using a TA Instruments DMA 2980 (TA Instruments, New Castle, DE, USA) in single-cantilever mode. Tests were performed with a 10 μm oscillation amplitude at 1 Hz while heating at 2 °C/min. Typical specimen dimensions were 35 mm× 12 mm × 3.2 mm.

Mechanical properties: Mechanical testing was performed at room temperature on an Instron 8872 (Instron, Norwood, MA, USA). Fracture toughness was measured following ASTM 5045-99 [22] using printed compact-tension specimens (15.5 mm × 14.88 mm × 5.5 mm) with an 8.7 mm notch. A sharp razor blade was used to initiate a pre-crack. Five to ten samples were tested per formulation at a crosshead speed of 0.5 mm/min. Flexural properties were measured using three-point bending according to ASTM D790 [23], with a span-to-depth ratio of 16:1 and specimen dimensions of 120 mm × 12.7 mm × 3.2 mm. Five samples were tested for each formulation.

Extent of cure: Attenuated total reflection (ATR) Fourier transform infrared (FTIR) spectroscopy in the mid-infrared (mid-IR) region was used to evaluate the extent of cure of printed and post-cured parts. The C=C of *AMP* and *PMMI* were characterized by peaks at 790 cm^−1^ and 690 cm^−1^, respectively. The fractional conversions, *α*, of *AMP* and *PMMI* double bonds were quantified using the equations below:

(1) $$\alpha_{AMP}=1-\frac{I_{790,cure\;stage}/I_{2850,cure\;stage}}{I_{790,\;resin}/I_{2850,\;resin}}$$

(2) $$\alpha_{PMMI}=1-\frac{I_{690,cure\;stage}/I_{2850,cure\;stage}}{I_{690,\;resin}/I_{2850,\;resin}}$$

where *I* is the height of the given peaks. The OCN (cyanate) group of the CE resin was characterized by the group of peaks at 2200–2300 cm^−1^. The reaction of CE was confirmed by the disappearance of the OCN group and the presence of the triazine ring, as evidenced by peaks at 1360 cm^−1^ and 1558 cm^−1^.

Density change upon curing: The density, *ρ*, of printed and post-cured parts was measured using a density gradient column following ASTM 1505-3 [24]. Samples were rinsed with deionized water before being placed in the column, and the sample position was recorded after 2 h of submersion. The density change was calculated using the following equation:

(3) $$\mathrm{density}\;\mathrm{change}=100\;\frac{(\rho_{cure\;stage}-\rho_{resin)}}{\rho_{resin}}$$

## 3. Results and Discussion

### 3.1. Selection of Resin and Exposure Time

**Figure 2:**
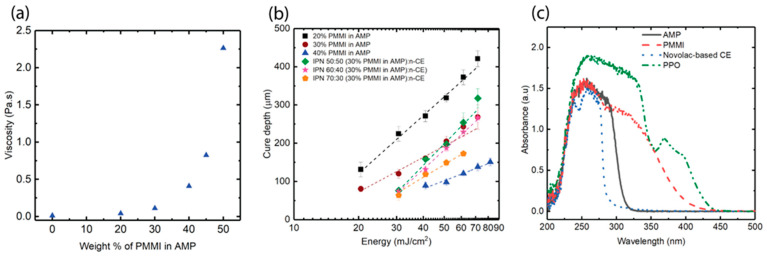
(**a**) Viscosity of *PMMI*-*AMP* resins vs. the weight percent of *PMMI* in resin, (**b**) working curves of all resins, (**c**) UV–vis spectra of *AMP*, *PMMI*, n-CE, and PPO.

Poly(phenylmethane) maleimide was selected because it dissolved well in *AMP* without recrystallization after cooling to room temperature from 70 °C. The viscosity of the resins containing different weight percentages of *PMMI* was investigated, as viscosity is one of the important processing parameters for vat photopolymerization. Figure 2a shows an exponential increase in viscosity with increasing weight percent of *PMMI*. The resins should have sufficiently low viscosity for printing, ideally below 0.5 Pa s. In addition, this low-viscosity *PMMI*-*AMP* resin will help dilute n-CE, which is a semi-solid at room temperature. Therefore, the resins containing more than 40 wt% of *PMMI* were not studied further.

Working curves were obtained to assess the depth of penetration (D_p_), critical energy (E_c_), and the appropriate print times for the investigated systems. Figure 2b shows the cure depth vs. energy on a semi-log plot for the *PMMI*-*AMP* resins and IPN resins, respectively. The energy is the product of power density and exposure time. The slope of the best-fit line to these data is D_p_, and the intercept is the product of -D_p_ln(E_c_), which allows the determination of E_c_. The D_p_ and E_c_ for all the resins are given in Table 1. As the *PMMI* concentration in the resin increases, its D_p_ decreases. The UV–vis spectra of the components (measurement method described in Supplementary Materials) show that *PMMI* also absorbs light at 405 nm together with PPO, while the remaining components in the resin do not absorb light at 405 nm (Figure 2c). Therefore, *PMMI* acts as a reactant as well as a light absorber, which contributes to the decrease in cure depth of the resins. With the *PMMI*-*AMP* resins, the lower the D_p_, the higher the required E_c_. The IPN resins require higher E_c_ due to the cyanate ester dilution effect. The *PMMI*-*AMP* resins were printed using 80 s per layer, except for the resin containing 40 wt% *PMMI* in *AMP*, for which 160 s was used. The same exposure time (80 s) was used for all the IPN resins to compare conversion, even though some *PMMI*-*AMP* systems could be printed adequately at lower exposure times.

### 3.2. Thermomechanical Properties of PMMI-AMP Systems

**Figure 3:**
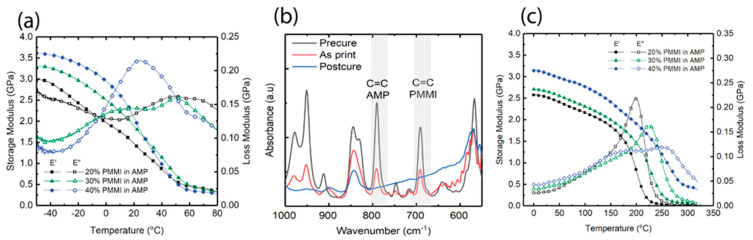
(**a**) DMA of *PMMI*-*AMP* resin samples after printing; (**b**) representative IR spectra of 20 wt% *PMMI* in *AMP* at different curing stages; (**c**) DMA of *PMMI*-*AMP* resin samples after thermal post-curing.

Dynamic mechanical analysis (DMA) was performed on printed and post-cured samples of *PMMI*-*AMP* systems. Figure 3a shows the storage modulus and loss modulus vs. temperature for the printed samples. The *T_g_* (based on storage modulus) is close to room temperature for all three systems. Therefore, the systems vitrify for the given photo-cure parameters at room-temperature reaction conditions. The loss moduli have a second peak at higher temperature, which can be attributed to continued reaction during DMA testing. Figure 3b shows representative FTIR spectra of the 20 wt% *PMMI*-in-*AMP* system. The conversion of *PMMI* and *AMP* was calculated according to Equations (1) and (2) and recorded in Table 2. A decrease in conversion of *PMMI* and *AMP* is observed as the concentration of *PMMI* increases for the printed samples. The conversion of *PMMI* for the 40 wt% *PMMI*-in-*AMP* system is significantly lower than that of the other two resins. This indicates the system reached vitrification at lower conversion, as is expected because *PMMI* possesses a higher *T_g_* than *AMP*.

The printed parts were then post-cured using the procedures described in the experimental section. The thermal post-cure procedures were divided into multiple temperature increments to prevent *AMP* evaporation. Figure 3c shows the DMA results for the post-cured samples. The storage moduli of the 20 and 30 wt% *PMMI*-in-*AMP* samples display rubbery behavior when heated up to 300 °C. On the other hand, the storage and loss modulus behavior of 40 wt% *PMMI* in *AMP* suggests that the reaction continues above 300 °C. MMI is known to require a high curing temperature to achieve a high crosslinking density [15]. Thus, at high *PMMI* concentrations (40 wt%), a higher curing temperature is required for full conversion. The experimental *T_g_* (E” peak) and *T_g_* estimated by using the Fox equation (Equation (4)) are given in Table 2.

(4) $$\frac{1}{T_{g}}=\frac{w_{1}}{T_{g1}}+\frac{w_{2}}{T_{g2}}$$

In this equation, *T_g_* is the glass transition temperature of the mixture, *w*_1_ and *w*_2_ are the weight fractions of the components, and *T_g_*_1_ and *T_g_*_2_ are the *T_g_* of the two components. The *T_g_* increases with an increase in concentration of *PMMI*. The *T_g_* of 20 wt% *PMMI* in *AMP* is used as a reference value to estimate the *T_g_* of *PMMI* using the Fox equation. Taking the *T_g_* of *AMP* polymer to be 150 °C, the estimated *T_g_* of *PMMI* is calculated to be 624 °C. The experimental *T_g_* of *PMMI*-*AMP* systems is lower than the estimated *T_g_*, but the difference is not significant. The DMA results and estimated *T_g_* are consistent with the conversion data shown in Table 2. There is unreacted *PMMI* in the 40% *PMMI*-in-*AMP* system, which results in a lower experimental *T_g_* than the estimated value.

It is observed that all *PMMI*-*AMP* blends are miscible and stable at room-temperature conditions. They all show promising characteristics, with high *T_g_*. Based on these results, the 30 wt% *PMMI*-in-*AMP* resin was selected to continue with the IPN study due to its intermediate properties, such as high *T_g_*, low viscosity, reasonable cure depth, and conversion of components during reaction.

### 3.3. Thermomechanical Properties of IPN Systems

**Figure 4:**
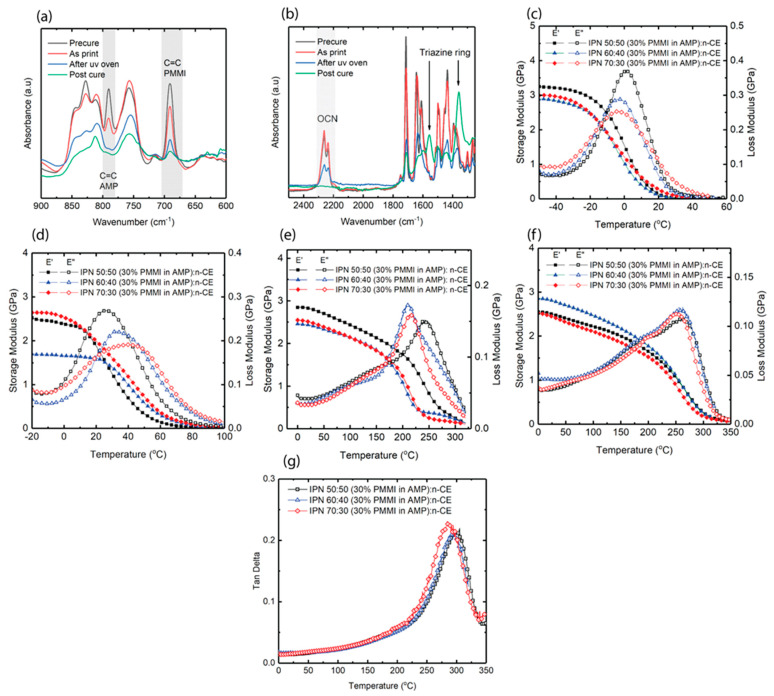
IR spectra of IPN 50:50 (30% *PMMI* in *AMP*):n-CE at curing stages: (**a**) C=C of *PMMI* and *AMP*; (**b**) -OCN group and triazine ring, DMA of IPN systems; (**c**) as-printed samples; (**d**) UV post-cured; (**e**) thermal post-cured; (**f**) second DMA of thermal post-cured samples; (**g**) tan delta of second DMA.

The resin of 30 wt% *PMMI* in *AMP* was selected for the IPN study as mentioned above. The IPN resins consisted of different weight percentages of n-CE to “30 wt% *PMMI* in *AMP*”. Since the novolac-based cyanate ester is semi-solid at room temperature, the *PMMI*-*AMP* dissolves the n-CE to obtain low-viscosity mixtures. The weight percent of n-CE used in this study did not exceed 50 wt% in order to keep the viscosity less than 3.0 Pa.s for printing. Figure 4a,b show the mid-IR spectra of IPN 50:50 (30% *PMMI* in *AMP*):n-CE at each curing stage. During printing, the co-polymerization of *PMMI* and *AMP* occurs, as reflected by changes in peak height at 690 cm^−1^ and 790 cm^−1^, respectively. On the other hand, cyanate ester does not react, as the group of peaks at 2200–2300 cm^−1^ remains unchanged. After UV post-curing, an additional significant amount of *PMMI* and *AMP* was found to react. Upon thermal post-curing, cyanate ester reacts and forms a triazine structure, associated with the appearance of peaks at 1360 cm^−1^ and 1558 cm^−1^.

Table 3 summarizes the conversions of reactive species attained at the various curing stages. The conversions of *PMMI* and *AMP* in as-printed IPN samples are higher than that of 30% *PMMI* in *AMP*, shown in the previous section. This indicates that in IPN systems, vitrification is delayed, which provides a less diffusion-limited environment for the reaction between *PMMI* and *AMP* to reach higher conversion. Figure 4c shows the DMA traces of as-printed IPN samples. The storage modulus at 25 °C is notably low, below 200 MPa, and the printed parts are observed to be soft and in the rubbery state. The *T_g_* by loss modulus of the as-printed samples is significantly lower than the cure temperature, which is room temperature. This is due to the plasticizing effect of unreacted cyanate ester in the network. After being UV-post-cured at 80 °C for 1 h, further photo-reaction occurs and can be observed from the IR conversion data and by the change in *T_g_*. Figure 4d shows that the *T_g_* increases significantly, from close to 0 °C to 26–43 °C. The storage modulus at 25 °C also increases to above 1 GPa. The C=C of *AMP* reaches full conversion in the IPN 50:50 (30% *PMMI* in *AMP*):n-CE system. For the other two formulations, *AMP* reaches conversion above 0.9. The conversion of *PMMI* also increases, but not as much as that of *AMP*, suggesting that homopolymerization of *AMP* is possible. Thermal post-curing induces cyanate ester to polymerize, as well as the rest of the *PMMI* and *AMP* in the system. The curing was performed in multiple temperature steps due to the exothermic reaction of cyanate ester [25,26]. Figure 4e,f show the first and second DMA runs of the thermal post-cured samples, respectively. Comparisons of these plots indicate that maleimide did not fully react during the post-curing step, as the mid-IR shows that cyanate ester fully reacted after post-cure in all cases; so the increase in *T_g_* is likely due to additional conversion of *PMMI* reactive groups. For 30 wt% *PMMI* in *AMP*, the previous section showed that *PMMI* almost reaches full conversion when cured to 280 °C; however, in the case of the IPN systems, we did not observe the same effect. This could be due to the presence of a fully cured cyanate ester network that creates a diffusion-limited environment for *PMMI* to fully react. Therefore, a higher curing temperature is needed for additional reaction, which was provided by the DMA temperature ramp to 320 °C. The storage moduli at room temperature of all samples increased compared with those before thermal post-curing. The *T_g_* was defined by the peak position of the loss modulus. The *T_g_* of IPN 50:50 (30% *PMMI* in *AMP*):n-CE is the highest as it has the highest cyanate ester content. All samples were tested on the DMA a second time to obtain the final *T_g_*, as *PMMI* continued to react when the samples were heated up to 320 °C in the first DMA. The final *T_g_* of all samples are close to each other as there is a compensation between having less cyanate ester but more maleimide. It is observed that the loss moduli of all systems are broad, with a secondary peak around 170 °C. However, the tan delta (Figure 4g) shows one single *T_g_* peak. Thus, the broad modulus could be due to network interaction rather than a second *T_g_*. The final *T_g_* was estimated using the Fox equation and is compared to the experimental results in Table 3. In Equation (4), *T_g_*_1_ is the experimental *T_g_* of 30% *PMMI*-in-*AMP* resin, 225 °C; *T_g_*_2_ is an estimated temperature of 325 °C for n-CE because n-CE has a broad *T_g_* ranging from 300 to 350 °C. The DMA of pure n-CE is provided in Figure S1 of the Supplementary Materials. With the estimated values used for n-CE, the calculated *T_g_* values were close to the experimental *T_g_* values. However, the *T_g_* of n-CE is reported to be as high as 400 °C [17], and the final *T_g_* that we obtained was not as high as expected. This is possibly due to competition between the crosslinking reaction and monomer degradation at high temperature. Figure 4g shows the tan delta of the second DMA run of the post-cured IPN systems. The *T_g_* (by tan delta peak) of the IPN 50:50 (30% *PMMI* in *AMP*):n-CE system is 300 °C, which is higher than any *T_g_* reported in the literature. The measured *T_g_* of IPN systems investigated and *T_g_* values estimated by the Fox equation are recorded in Table 3.

### 3.4. Mechanical Properties of IPN vs. PMMI-AMP Systems

**Figure 5:**
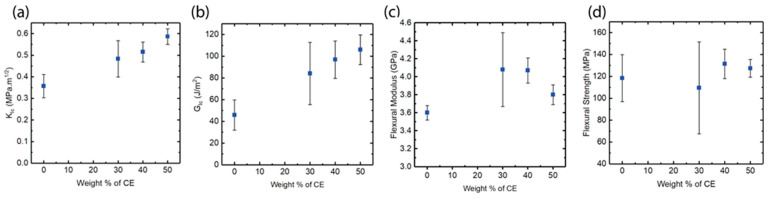
Mechanical properties of IPNs vs. 30 wt% *PMMI* in *AMP*: (**a**) stress intensity factor, K_Ic_; (**b**) critical strain energy release rate, G_Ic_; (**c**) flexural modulus; (**d**) flexural strength.

IPN polymer architectures are widely used to toughen materials. The fracture toughness of the IPNs was tested and compared with the toughness of 30 wt% *PMMI* in *AMP*. Figure 5a,b show the stress intensity factor (K_Ic_) and the critical strain energy release rate (G_Ic_) of the systems relative to CE content. MMI is known to be brittle because of its high crosslinking density. Therefore, a low fracture toughness of *PMMI*-*AMP* is expected. The IPNs have significantly higher fracture toughness compared to the *PMMI*-*AMP* system. This observation is in agreement with reports in the literature that IPN network architectures result in toughened systems [27,28,29]. The reported G_Ic_ of pure novolac-based cyanate ester is 60 J/m^2^ [16]. A synergistic effect is observed as the resulting network possesses higher toughness than the individual networks. The values of G_Ic_ and K_Ic_ increase with increasing CE concentration. The sample with 50 wt% CE has the highest toughness, with G_Ic_ of 106 J/m^2^. The G_Ic_ of other IPNs are also relatively high for a high-temperature system as most high-temperature systems are brittle due to high crosslinking density.

Figure 5c,d show the flexural strength and flexural modulus of the IPNs with varying amounts of n-CE and the 30 wt% *PMMI*-in-*AMP* control sample. Overall, all formulations possess excellent flexural properties. In all cases, samples have a flexural strength greater than 100 MPa and flexural modulus greater than 3.5 GPa. The IPN systems have a significantly higher flexural modulus than the pure *PMMI*-*AMP* system, with a maximum value higher than 4 GPa for systems containing between 30 and 40 wt.% CE. The flexural strength is higher for the IPN systems, but there is no distinct trend with the increase in n-CE concentration. The standard deviation for IPN 70:30 (30% *PMMI* in *AMP*):n-CE is higher than the others and may be attributed to sample defects. Thus, the IPNs exhibit good mechanical properties in addition to high *T_g_*_,_ and their performance is significantly better than that of the corresponding pure 30% *PMMI* in *AMP*.

### 3.5. Density Change upon Curing

The density and the percent change in density are given in Table 4 for samples at different curing stages. The density of thermosets changes during reaction due to the formation of covalent bonds. It is observed that the overall density change in the IPN systems is lower than that in the *PMMI*-*AMP* system. The IPN also exhibits lower overall shrinkage after post-cure. As expected, the change in density is also inversely proportional to the concentration of CE. It is known that CE possesses very low cure shrinkage, and as a result, the more CE that is used for the IPN, the less overall cure shrinkage is observed. The observed lower shrinkage of the IPN and low modulus after printing could potentially reduce the residual stresses of the printed part. For the IPN systems, the relatively low change in density observed upon UV post-curing in the rubbery state, compared to that during printing, suggests that the gelled state attenuates cure shrinkage associated with double-bond conversion. Interestingly, the change in density between print and post-cure of the *PMMI*-*AMP* sample was much lower than the change in conversion would suggest. This could be due to mass loss during curing; however, the mass loss is below 2%. The mass loss before and after post-cure of samples is provided in Table S1 of the Supplementary Materials. An alternative explanation is that vitrification at a lower temperature imposes physical constraints on shrinking. Nevertheless, the explanation of the observed phenomenon poses an open question that merits further investigation.

### 3.6. Printed Part Fidelity

**Figure 6:**
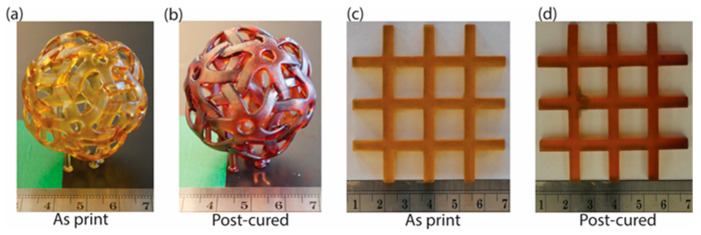
Parts printed using resin IPN 50:50 (30% *PMMI* in *AMP*):n-CE: (**a**) as-printed fullerene ball; (**b**) post-cured fullerene ball; (**c**) as-printed grid, 60 mm × 60 mm × 4 mm overall size; (**d**) post-cured grid.

Given that thermal and mechanical properties, conversion, and changes in density have been discussed, it is crucial to demonstrate next that the developed resins can produce parts with high fidelity. Although the as-printed IPN parts have a low modulus at room temperature and are in a rubbery state, they maintain their shape and dimensions after post-curing. Figure 6 shows some geometries printed with IPN 50:50 (30% PMMA in *AMP*):n-CE before and after post-cure. Figure 6a,b demonstrate that the IPN formulations can be used to print complex and detailed geometries, and that the shapes of the printed parts remain unchanged after post-curing.

To quantify the dimensional change before and after post-curing, a grid with an overall size of 60 mm × 60 mm × 4 mm and a post width of 4 mm was printed (Figure 6c) and post-cured (Figure 6d). The widths of all the vertical and horizontal posts, as well as the overall thickness, were measured. The average printed post width was 4.05 ± 0.03 mm, 1.27% larger than the input model. This can be attributed to the resin’s relatively high D_p_. The lower the D_p_, the better the resolution. Moreover, this discrepancy can be mitigated by slightly modifying the print model to achieve the desired dimensions. More importantly, after the grid was fully post-cured, the change in size was less than 0.5% in all dimensions. This significant result confirms that these IPN resins can provide parts with high thermal and mechanical properties that retain their shape and dimensions after post-curing at high temperatures.

### 3.7. Control of Print Conversion

**Figure 7:**
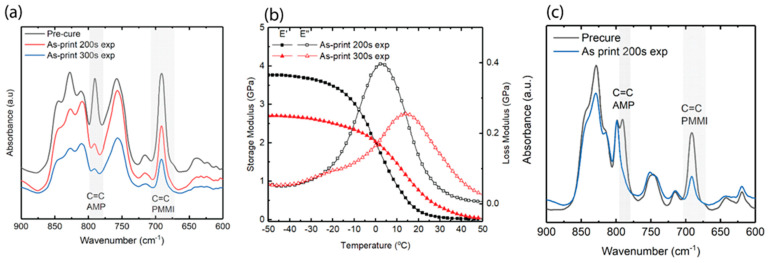
(**a**) DMA of as-printed IPN 50:50 (30% *PMMI* in *AMP*):n-CE using 200 s and 300 s exposure time per layer; (**b**) IR spectra of as-printed IPN 50:50 (30% *PMMI* in *AMP*):n-CE using 200 s and 300 s exposure time per layer; (**c**) IR spectra of as-printed IPN 50:50 (30% *PMMI* in *AMP*):bisE-CE using 200 s exposure time.

A typical vat photopolymerization process happens at room temperature. Therefore, the reaction becomes diffusion-limited when it reaches vitrification at the reaction temperature. *PMMI*-*AMP* resins demonstrate this. In the sequential IPN used in this study, unreacted cyanate ester delays early vitrification, leaving the part in the rubbery state with a low modulus. Based on the results from previous sections, the IPN 50:50 (30% *PMMI* in *AMP*):n-CE resin was selected for this conversion study because it has the highest n-CE concentration. The resin was printed with exposure times of 200 and 300 s per layer and a layer thickness of 100 microns. The IR spectra show a significant decrease in the peak at 790 cm^−1^ for *AMP* (Figure 7a). The DMA indicates that *T_g_* increases but remains below room temperature (Figure 7b). As established in the previous section, *AMP* can reach full conversion with a combination of higher temperature and additional light exposure with the photo post-cure step. Despite higher energy input from increased exposure time, the photo-reaction was still curtailed by diffusion limitations. To achieve full conversion of at least the *AMP* moieties at room temperature, n-CE was replaced with a low-viscosity bisphenol E cyanate ester. The ratio of bis-CE to 30% *PMMI* in *AMP* was kept at 50:50, and printing was conducted using a 200 s exposure time per layer. With this approach, it is observed that *AMP* reaches full conversion, and *PMMI* conversion improves to 0.65 (Figure 7c).

### 3.8. Bonding Between Layers

**Figure 8:**
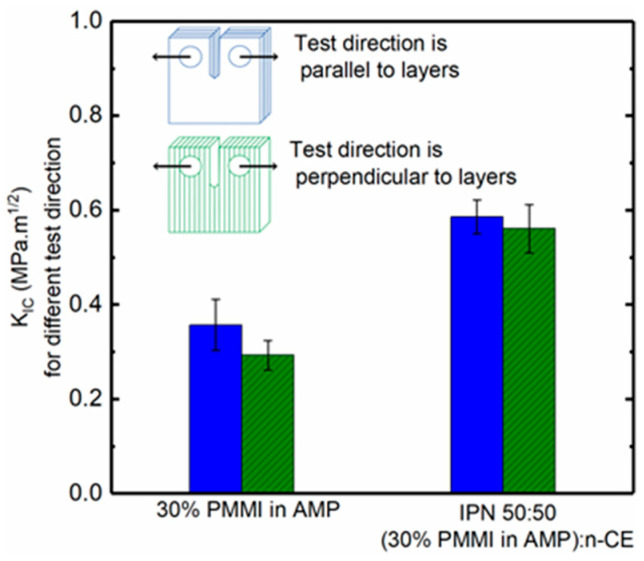
K_Ic_ for samples of 30% *PMMI* in *AMP* and IPN 50:50 (30% *PMMI* in *AMP*):n-CE where test direction is perpendicular or parallel to layer orientation.

During vat photopolymerization additive manufacturing, a layer bonds to the previously deposited layer because the material is partially cured, and the cure depth exceeds the layer thickness. With the in situ sequential IPN, we hypothesize that additional bonding between layers is provided by CE reaction during post-curing. This hypothesis was tested using compact tension tests on fully post-cured samples. The compact tension samples were printed in two directions, where the layers were either parallel or perpendicular to the test direction. The 30% *PMMI*-in-*AMP* resin and the IPN 50:50 (30% *PMMI* in *AMP*):n-CE resin were selected to test the hypothesis. Figure 8 shows the K_Ic_ values for different test directions, and the average values of G_Ic_ and K_Ic_ are summarized in Table 5. The average values show only a slight difference in fracture toughness of the 30% *PMMI*-in-*AMP* system when printed with different orientations, and the toughness values of the IPN are close to each other as expected. Surprisingly, in both cases, the difference in toughness with respect to orientation is not statistically significant. The reason for this is that while the IPN system has a second network for additional bonding between layers upon post-cure, the 30 wt% *PMMI* in the *AMP* system has low conversion after printing, allowing for further reaction during thermal post-curing.

## 4. Conclusions

This investigation demonstrated that high-performance thermosetting materials can be printed using vat photopolymerization by formulating in situ sequential IPN of *PMMI* and CE. This technique is possible due to the copolymerization reaction between *PMMI* and *AMP* that occurs during printing, resulting in a cyanate ester-swollen network with a sub-room-temperature *T_g_*. Cyanate ester polymerization occurs during the high-temperature post-printing step. During printing, vitrification is delayed by the presence of unreacted second-network cyanate ester monomers. The resulting materials possess a *T_g_* above 250 °C, as measured by the loss modulus peak position, and improved toughness. The density changes upon curing are also reduced due to a lower double-bond concentration and the presence of a low-cure-shrinkage cyanate ester resin system. The IPN formulations developed in this work can provide parts with high fidelity, as the shape and dimensions of printed parts are maintained after thermal post-curing, which is used to attain the very high *T_g_*.

## Figures and Tables

**Table 1:** D_p_ and E_c_ of *PMMI*-*AMP* resins and IPN resins.

Resin	D_p_ (μm)	E_c_ (mW/cm^2^)
20% *PMMI* in *AMP*	223.3	11.5
30% *PMMI* in *AMP*	152.6	13.7
40% *PMMI* in *AMP*	93.8	16.7
IPN 50:50 (30% *PMMI* in *AMP*):n-CE	272.5	23.5
IPN 60:40 (30% *PMMI* in *AMP*):n-CE	249.8	20.7
IPN 70:30 (30% *PMMI* in *AMP*):n-CE	169.8	14.7

**Table 2:** Conversion of *PMMI* and *AMP* at curing stages and experimental and estimated *T_g_* of *PMMI*-*AMP* systems.

	Cure Stage	20% *PMMI* in *AMP*	30% *PMMI* in *AMP*	40% *PMMI* in *AMP*
*α_AMP_*	As printed	0.48	0.46	0.41
	Post-cured	1	1	1
*α_PMMI_*	As printed	0.29	0.24	0.09
	Post-cured	1	0.98	0.91
*T_g_* (°C)	Post-cured	200	225	252
*T_g_* (°C)	Estimated (Fox)	Used as reference	230	263

**Table 3:** Conversion of *AMP*, *PMMI*, and CE at curing stages and the *T_g_* of the systems.

	Cure Stage	IPN 70:30 (30% *PMMI* in *AMP*):n-CE	IPN 60:40 (30% *PMMI* in *AMP*):n-CE	IPN 50:50 (30% *PMMI* in *AMP*):n-CE
*α_AMP_*	As printed	0.61	0.53	0.63
	UV post-cure	0.91	0.96	~1
	Thermal post-cure	1	1	1
*α_PMM_*_I_	As printed	0.32	0.27	0.28
	UV post-cure	0.4	0.41	0.42
	Thermal post-cure	0.87	0.85	0.89
α_n-CE_	As printed	0	0	0
	UV post-cure	0	0	0
	Thermal post-cure	1	1	1
*T_g_* (°C)	Thermal post-cure	216	212	245
	Second DMA (E”)	259	260	266
	Tan delta	287	294	300
	Estimated (Fox)	251	260	270

**Table 4:** Density and percent change in density for samples at curing stages.

	Cure Stage	30% *PMMI* in *AMP*	IPN 70:30 (30% *PMMI* in *AMP*) :n-CE	IPN 60:40 (30% *PMMI* in *AMP*) :n-CE	IPN 50:50 (30% *PMMI* in *AMP*) :n-CE
ρ (g/cm^3^)	Resin	1.1748	1.2018	1.2073	1.2151
	As printed	1.2757	1.2616	1.2601	1.2757
	UV post-cured	1.2785	1.2781	1.2817	1.2829
	Thermal post-cured	1.2817	1.2841	1.2838	1.2853
% change in density	As printed vs. resin	8.58	4.95	4.37	4.98
	UV post-cured vs. resin	8.82	6.34	6.16	5.58
	Thermal post-cured vs. resin	9.1	6.84	6.34	5.77

**Table 5:** G_Ic_ and K_Ic_ of fully post-cured samples printed at different orientations.

	30% *PMMI* in *AMP*		IPN 50:50 (30% *PMMI* in *AMP*):n-CE	
	Layers Perpendicular to Test Direction	Layers Parallel to Test Direction	Layers Perpendicular to Test Direction	Layers Parallel to Test Direction
G_Ic_ (J/m^2^)	45.8 ± 13.9	30.0 ± 6.4	106.0 ± 13.7	99.0 ± 18.0
K_Ic_ (MPa.m^1/2^)	0.357 ± 0.054	0.293 ± 0.031	0.586 ± 0.036	0.561 ± 0.051

## Data Availability

The data presented in this study are available on request from the corresponding author.

## Supplementary Materials

The following supporting information can be downloaded at: https://www.mdpi.com/article/10.3390/polym17233179/s1, Figure S1: DMA of pure n-CE; Table S1: Percent mass change between as-print vs. post-cured sample.

## Abbreviations

*AMP*: acryloylmorpholine; MMI: multimaleimide; *PMMI*: poly (phenylmethane) maleimide; bisE-CE: bisphenol E cyanate ester; n-CE: novolac-based cyanate ester; IPN: interpenetrating polymer network; DMA: dynamic mechanical analysis; FTIR: Fourier transform infrared, G_Ic_: critical strain energy release rate; K_Ic_: stress intensity factor; SLA: stereolithography additive manufacturing; DLP: digital light processing.
