# Supplementary material for: High-Tg Vat Photopolymerization Materials Based on In Situ Sequential Interpenetrating Polymer Networks of Maleimide and Cyanate Ester Monomers
Source: Polymers (Basel). 2025 Nov 29;17(23):3179. doi: 10.3390/polym17233179 (PMC12694268; doi:10.3390/polym17233179)
Supplement: Supplementary file 1 [file polymers-17-03179-s001.zip › polymers-3991087-supplementary.pdf]

# Supporting Information

# High $T_g$ Vat Photopolymerization Materials Based on *In-situ* Sequential Interpenetrating Polymer Networks of Maleimide and Cyanate Ester Monomers

Anh Fridman[a], Nicolas J. Alvarez[a], Giuseppe R. Palmese[a,b,*]

[a] Department of Chemical and Biological Engineering, Drexel University, Philadelphia, PA 19146, USA

[b] Department of Chemical Engineering, Rowan University, Glassboro, NJ 08028, USA

### UV-Vis Measurement

The absorbance of individual components in the resins was measured using Ocean Optics UV-Vis. Each chemical was dissolved in acetonitrile (Sigma Aldrich) at the concentration of 0.1 wt%. The solution was measured using a quartz cuvette.

### Tg of CE using DMA

600 ppm of Copper (II) napthenate was added to novolac-based cyanate ester as described in Experimental section. The mixture was cured in a silicone mold at 120 °C for 2 hrs, 180 °C overnight, 220 °C for 3 hrs, 280 °C for 1 hr, and 300 °C for 30 minutes. Dynamic Mechanical Analysis was performed on the cured sample to find out the $T_g$ of the pure n-CE. **Figure S1** shows the storage modulus and loss modulus vs. temperature for the pure n-CE. The loss modulus is broad, with multiple peaks. It is known for its high $T_g$ and broad loss modulus peak. To estimate the $T_g$ of the IPN, using the Fox equation we selected the last peak at around 325 °C as the $T_g$ of pure n-CE.

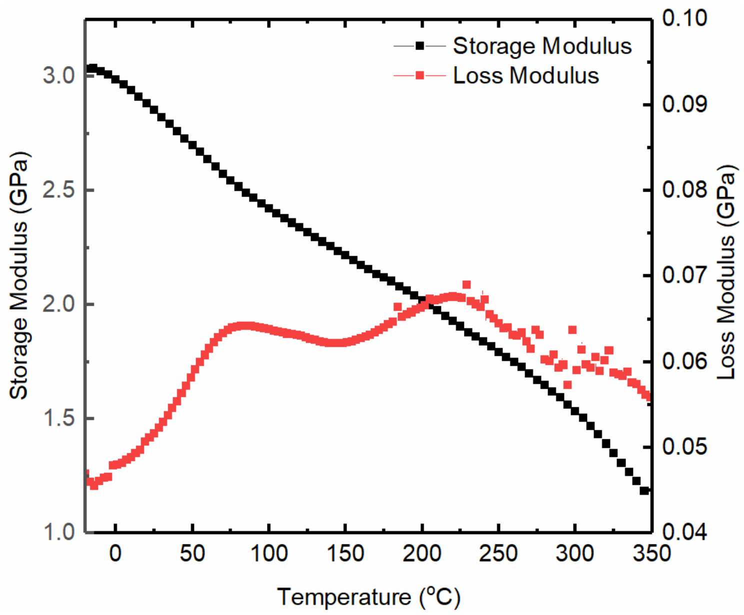


**Figure S1**: DMA of pure n-CE

**Mass Loss of Samples Upon Post-Curing**

The mass of printed samples with various PMMI amounts in AMP was measured before and after post-curing. The percentage change in mass before and after post-curing is found in Table S1. There was minimal mass loss during curing.

**Table S1: Percent mass change between as-print vs. post-cured sample**

| | % mass change between print vs. post-cured sample |
|---|---|
| 20% PMMI in AMP | 1.29 |
| 30% PMMI in AMP | 1.97 |
| 40% PMMI in AMP | 0.34 |
